# Ten-year review of ST-Segment Elevation Myocardial Infarction (STEMI) in Tanzania: a single center retrospective review

**DOI:** 10.11604/pamj.2024.49.82.45351

**Published:** 2024-11-18

**Authors:** Nadeem Kassam, Mohamed Varwani, Mzee Ngunga, Mohamed Jeilan, Mangaro Mabusi, James Orwa, Salim Surani, Robert Mvungi, Nasiruddin Jamal

**Affiliations:** 1 Section of Cardiology, Aga Khan University Hospital, Nairobi, Kenya,; 2 Section of Cardiology, Aga Khan Hospital, Dar es Salaam, Tanzania,; 3 Department of Population Health, Aga Khan University Hospital, Nairobi, Kenya,; 4 Department of Medicine, Aga Khan University Hospital, Nairobi, Kenya

**Keywords:** Segment elevation myocardial infarction, Tanzania, ischemic heart disease, in-hospital mortality, 10-year review

## Abstract

**Introduction:**

Ischemic Heart Disease (IHD) is an emerging epidemic in sub-Saharan Africa (SSA). Although the true burden may be underreported in the African continent, it still remains one of the leading causes of death among adults aged above 60 years. ST-Segment Elevation Myocardial Infarction (STEMI) is a clinically time-sensitive fatal sequela of IHD with timely reperfusion by primary Percutaneous Coronary Intervention (PCI) being the gold standard of care. There has been steady progress in coronary care services in Tanzania, alongside a rise in IHD-related risk factors. However, data on this is limited. This study aimed to examine trends in STEMI over the past decade and identify factors associated to in-hospital mortality.

**Methods:**

this single-center retrospective study was conducted at the Aga Khan Hospital Dar-es-Salaam (AKHD), Tanzania. The AKHD is one of the pioneers in establishing the first cardiac catheterization laboratory in the nation. The current study involved extracting relevant data of all patients who presented with STEMI from August 2014 to December 2023. Descriptive statistics were used to define the population. Patient´s outcomes were based on hospital survival. Binary logistic regression was run (at 95% CI and p-value<0.05) to identify the determinants for in-hospital mortality.

**Results:**

two hundred and thirty (n=230) patients were included in the final analysis. The cohort was predominantly male (83.5%, n=192), with a median age of 55.0 years (IQR 48.0-65.0). More than half of the cohort were patients with Diabetes (56.9%, n=131) and hypertension (51.6%, n=111), presenting in Killip class I symptoms (54.3%, n=125). Most patients presented with chest pain (n=162,72.6%), with a median duration of 12.2 hours (IQR 3.0-24.0 hours). The left anterior descending (LAD) artery was the culprit vessel in most cases (48.7%, n=112). A total of 163 (70.8%) patients underwent Primary-PCI. A mean BMI above 36.2 kg/m^2^(±5.7) (OR 1.46, CI 1.17-2.10), the presence of smoking (OR 41.68, CI 2.60-240.71), and the need for mechanical ventilation (OR 77.42, CI 1.95-128.89) were factors associated with in-hospital mortality.

**Conclusion:**

the in-hospital mortality among patients with STEMI at our hospital was 5.7%. Cigarette smoking, obesity and the need for mechanical ventilation were predictors of poor in-hospital outcomes.

## Introduction

Cardiovascular disease (CVD) is an emerging epidemic in sub-Saharan Africa (SSA) and other low- to middle-income countries (LMICs) like Tanzania [1]. Cardiovascular disease accounts for approximately 13% of all deaths in Tanzania, higher than in most African countries [2]. Various reports have speculated that the true burden of ischemic heart disease (IHD) is likely underestimated in the majority of African countries due to limited awareness, insufficient clinician training, and lack of resources, yet it still accounts for the single most common cause of cardiovascular mortality worldwide [3]. Economic growth and lifestyle changes in SSA over recent years have led to an increase in IHD risk factors, particularly among younger populations [4]. This has resulted to an increased burden of health expenditures across many sub-Saharan regions. Understanding the trend and the epidemiological transformation, as well as improving measures to stem the global tide associated with CVD mortality, remains an important action frontier in these regions [5].

ST-Segment Elevation Myocardial Infarction (STEMI) is a clinically time-sensitive fatal sequela of ischemic heart disease (IHD) [6]. The treatment is timely reperfusion by percutaneous coronary intervention (PCI) or thrombolytic therapy of the culprit coronary artery [7]. There has been a notable decrease in the mortality rate associated with ST-segment elevation myocardial infarction (STEMI) in resourceful High-Income Countries (HICs), mostly due to overall improvement in healthcare infrastructure and systems of care [8]. This may not be true for the majority of the sub-Saharan region. The impact of IHD remains a major public health burden due to various factors; including insufficient health care systems, lack of resources, skewed allocation of budget, high cost of treatment coupled with lack of health care professionals able to emulate its timely and ideal care [9].

To date, there is a paucity of available data on the state of coronary care within most LMICs in sub-Saharan Africa. In the past decade, Tanzania has witnessed a gradual and continued development in the ability to provide coronary care in both the private and public sectors. Simultaneously, recent national trends also portray an increased magnitude of multiple risk factors associated with coronary artery disease in Tanzania. In light of the country now experiencing a shift in its health landscape, the traditional focus on infectious disease is gradually giving way to the rising burden of non-communicable diseases (NCDs) with cardiovascular diseases assuming a prominent role; this analysis is timely [10].

The rationale for the study stems from the increasing prevalence of Ischemic Heart Disease in the sub-Saharan region (IHD) including Tanzania, where these conditions remain underreported and poorly understood. This observational study aimed to add to the already existing knowledge by analyzing demographics, clinical presentations, angiographic findings, and patient outcomes related to STEMI at a leading private hospital in Tanzania. Findings from the study can ultimately assist resource allocation, improve strategies, identify prognostic factors, and generate interventional targets for a region battling the tide of IHD.

## Methods

**Study design and setting:** this was a single-center retrospective study conducted at the Aga Khan Hospital Dar-es-Salaam (AKHD), Tanzania. AKHD is a private, non-profit, tertiary hospital in Tanzania. The AKHD was the first hospital in the country to have a 24-hour operational catheterization laboratory and the only hospital to date in Tanzania to be accredited by the Joint Commission International (JCI). The catherization laboratory was operational from 2014. The accreditation by JCI attests to quality patient care guided by evidence-based practice and continuous monitoring of clinical outcomes. The AKHD is a teaching hospital for the Aga Khan University Medical College, East Africa (Dar-es-Salaam Campus). The coronary care unit (CCU) of the AKHD is a 4-bed unit able to provide level III care [11]. Patients admitted to the unit receive 1: 1 nursing care. There is 24-hour coverage of the unit by a registered medical officer and on-call interventional cardiologist.

**Study population:** the hospital's coronary care unit registry, which contains data on all patients admitted to the unit, was used to identify patients who presented with STEMI throughout the study period.

**Inclusion criteria:** the current study involved extracting data from charts and electronic medical records for patients who presented with STEMI from August 2014 to December 2023. Patients 18 years and older who presented with STEMI as per the third and fourth universal definitions of myocardial infarction were included in the study [12].

**Exclusion criteria:** patients with incomplete medical records, duplicate medical records, alternate diagnoses such as myopericarditis, Takotsubo cardiomyopathy, and those who were transferred out or those who left against medical advice were excluded from the final analysis.

### Study variables

**Dependent variables:** final hospital outcome, length of hospital stay, need for organ support, left ventricular function, cause of acute coronary syndrome (ACS).

**Independent variables:** demographics, comorbidities and risk factors, clinical presentation, biochemical parameters changes, coronary angiography findings, type of intervention performed, and medical therapy received.

**Data collection:** data collected included multiple variables such as: demographics, comorbidities and risk factors, clinical presentation, biochemical parameters, electrocardiogram (ECG) changes (territory involved), coronary angiography findings (number of vessels, culprit lesion, and suspected mechanism of ACS), intervention performed, medical therapy received, the need of organ support, length of hospital stay and final hospital outcome. Patients were followed up to hospital discharge and grouped as survivors and non-survivors. Interventional cardiologists determined the cause of ACS according to previously validated methods [13,14] and were grouped into atherosclerotic [14], thrombotic [14], or spontaneous coronary artery dissection [13,14]. Obstructive Coronary Artery Dissection (CAD) was defined by invasive coronary angiography as a narrowing of the internal diameter >50% stenosis of the left main stem and >70% stenosis in a major coronary epicardial vessel [15]. A percentage diameter less than the one mentioned above was characterized as non-obstructive coronary artery disease [16]. Left ventricular function on 2D echocardiography performed during admission was documented and confirmed by the attending cardiologist. At the AKHD, all patients are managed with either one of the following strategies: a) primary Percutaneous Coronary Intervention (PCI); b) rescue PCI; c) conservative management in those presenting with a fully evolved myocardial infarct; and d) thrombolysis when primary PCI services aren´t available. Patients who have undergone thrombolysis on or off-site generally undergo a pharmaco-invasive approach. All data, both in paper form and electronic format, was collected by the primary investigator and checked by the supervising faculty for accuracy and completeness.

**Data analysis:** the collected data was incorporated into a Microsoft Excel 2010 (Redmond, WA, USA). Categorical data were reported as frequencies and proportions and compared with Pearson chi-square or Fisher´s exact tests. Continuous variables were reported as means or medians and compared with students' t-tests or the Wilcoxon rank-sum test. Univariable and multivariable logistic regression analyses were used to determine the predictors of in-hospital all-cause mortality. Any variable demonstrating statistical or clinical significance in explaining ICU mortality was considered in the multivariate model. We presented the adjusted odds ratios with their 95% confidence intervals (95% CI). Statistical significance was considered at a p-value < 0.05. Analysis was performed using Stata version 17 (StataCorp Ltd., College Station, TX, USA).

**Ethical clearance:** the study was approved by the Aga Khan University, East Africa Ethical Research Committee (AKU, EA ERC). The National Institute for Medical Research (NIMR) in Tanzania mandates the AKU, EA ERC to approve health research conducted by Tanzanian students. The hospital´s ethical committee and the AKU, EA, and ERC exempted the primary investigator from acquiring informed consent from the study participants since the study design did not affect the rights and welfare of the patients. This study was conducted in accordance with the Declaration of Helsinki. Ethical Reference number (AKU/2023/018/fb/04/02).

## Results

We identified 351 from medical records; after careful verification and search of records, 121 patients were excluded due to missing medical records, duplication, or alternative diagnosis (high-risk NSTEMI) and staged PCI procedures entry. 230 patients were included in the final study analysis.

**General characteristics of the study population:** the demographic and clinical characteristics of the patients are summarized in Table 1. The cohort was predominantly male (83.5%, n=192), with a median age was 55.0 years (IQR 48.0-65.0). The majority of the patients were aged between 45-60 years (50.8%, n=117), had underlying diabetes mellitus (56.9%, n=131), hypertension (51.6%, n=111) and were on treatment with cholesterol-lowering medication before presentation (60.5%, n=160). Most patients presented with chest pain (72.6%, n=162), with the median duration of chest pain before hospital presentation of 12.2 hours (IQR 3.0-24.0 hours). Most patients presented in Killip class 1 (54.3%, n=125). Anterior myocardial infarction on ECG was the most common presentation (59.1%, n=136). A small fraction of patients underwent thrombolysis prior to intervention (7.8%, n =17) as seen in Table 2.

**Table 1:** baseline demographics of the cohort

Characteristics	n	N = 230^1^
Median age	230	55.0 (48.0 - 65.0)
**Age group, n (%)**		
< 45 years		34 (16.0)
45-60		117 (50.8)
> 60 years		79 (37.1)
**Ethnicty, n (%)**	**230**	
African		80 (34.7)
South Asian		101 (43.9)
Other		40 (17.4)
Caucasian		9 (3.9%)
**Sex, n (%)**	**230**	
Female		38 (16.5)
Male		192 (83.5)
BMI (Kg/m^2^), median (IQR)	230	26.5 (25.0 – 31.0)
**BMI category**		
Normal		49 (21.3)
Overweight		79 (34.3)
Obesity I		55 (23.9)
Obesity II		32 (13.9)
Obesity III		15 (6.5)
**Admitting category, n (%)**	**230**	
Referral		85 (37.0)
Self		135 (63)
**Atherosclerotic risk factors**		
DM, n (%)		131 (56.9)
HTN, n (%)		111 (51.6)
Family history of premature ASCVD, n (%)		64 (29.8)
CKD/ESRD, n (%)		27 (12.6)
Previous ASCVD, n (%)		52 (24.2)
Smoking, n (%)		72 (33.5)
On cholesterol- lowering medication prior presentation, n (%)		130 (60.5)

1 : median (IQR); n (%); BMI: body mass index; DM: diabetes mellitus; HTN: hypertension, CKD: chronic kidney disease; ESRD: end stage renal disease; ASCVD: atherosclerotic cardiovascular disease

**Table 2:** symptoms and presentation of the cohort

Characteristic	N = 230^1^
**Presenting complains, n (%)**	
Chest pain	167 (72.6)
Cardiac arrest on presentation	4 (1.7)
Dyspeptic syndrome	12 (5.2)
Dyspnea	41 (17.8)
Syncope	6 (2.6)
Chest pain duration, (IQR)	12.2 hours (IQR 3.0 - 24.0)
<3 hours, n (%)	39 (23.1)
3-12 hours, n (%)	25 (14.9)
12-24 hours, n (%)	22 (13.4)
>24 hours, n (%)	81 (48.5)
**Killip, n (%)**	
Killip I	125 (54.3)
Killip II	59 (25.6)
Killip III	32 (13.9)
Killip IV	14 (6.1)
**ECG changes, n (%)**	
Anterior - septal	67 (29.1)
Inferior	59 (25.7)
Anterior- lateral	41 (17.8)
Anterior	28 (12.2)
Inferior- lateral	18 (7.8)
Lateral	8 (3.5)
Complete heart block	5 (2.2)
Posterior- lateral	4 (1.7)
Thrombolysis, n (%)	16 (7.8)
Off-site	12 (75)
On-site	4 (25)

1 : n (%); ECG: electrocardiogram

**Coronary angiography findings and interventions:** angiographic findings and interventions performed are illustrated in Table 3. The majority of the patients underwent a femoral puncture (61.7%, n=142), with single vessel disease (65.6%, n=151) and atherosclerosis (79.1%, n=182) as the most common finding and mechanism of obstruction. Left anterior descending (LAD) artery was the culprit vessel in most cases (48.7%, n=112). A total of 163 (70.8%) patients underwent Primary Percutaneous intervention (PCI).

**Table 3:** angiographic findings and interventions

Characteristics	n	N=230^1^
**Access. n (%)**	**230**	
Radial		88 (38.2)
Femoral		142 (61.7)
**Angiographic findings, n (%)**	**230**	
Non-obstructive coronary		17 (7.9)
Single vessel disease		142 (61.7)
Single vessel-ISRS		5 (2.2)
Single vessel-stent thrombosis		4 (1.7)
Two vessel disease		37 (16.1)
Triple vessel disease		25 (10.9)
**Culprit vessel, n (%)**	**230**	
LM		2 (0.87)
LAD		112 (48.7)
RCA		69 (30)
LCX		32 (13.9)
N/A		17 (7.9)
**Intervention done, n (%)**	**230**	
Coronary angiography-no intervention		17 (7.4)
Primary PCI		126 (54.7)
Thrombotic aspiration + primary PCI		12 (5.2)
POBA		7 (3.04)
Primary PCI + PCI of non-IRA		25 (10.9)
Rescue PCI		15 (6.5)
Coronary angiography - referral for CABG		17 (7.4)
Unsuccessful PCI		11 (4.8)
**Mechanism**	**230**	
Atherosclerotic		182 (79.1)
Thrombotic		29 (12.6)
SCAD		2 (0.87)
N/A		17 (7.4)

1 : n (%); LM: left main; LAD: left anterior descending; RCA: right coronary artery; LCX: left circumflex; PCI: percutaneous coronary intervention; SCAD: spontaneous coronary artery dissection; IRA: infarct-related artery; CABG: coronary artery bypass graft; ISRS: In Stent ReStenosis

**Clinical comparison of survivors and non-survivors:** the in-hospital mortality of the cohort was 5.7%. Patients who died prior to discharge were more likely to have diabetes mellitus (92.3%, n=12), hypertension (92.3%, n=12), a history of current or previous smoking (84.6%, n=11), and to be on treatment for hyperlipidemia (76.9%, n=10) (P-value <0.05). Additionally, an increased need for mechanical ventilation (30.8%, n=4) and inotropes (61.5%, n=8) was also noted amongst the non-survivors with statistical significance (P-value <0.05) as seen in Table 4.

**Table 4:** comparison of survivors and non-survivors

Characteristic	Overall, N = 230^1^	Survivors, N = 217 (94.3%)	Non- Survivors, N = 13 (5.7%)	P-value^2^
Age, median (IQR)	55.0 (48.0 - 65.0)	54.1 (48.0 - 65.0)	55.5 (49.0 - 75.0)	0.77
**Sex, n (%)**				**0.99**
Female	38 (10.7)	37 (10.9)	1 (7.7)	
Male	192 (89.3)	180 (89.1)	12 (92.3)	
Chest pain duration, median (IQR)	12.0 (3.0 - 24.0)	10.5 (3.0 - 24.0)	24.0 (24.0 - 36.0)	0.05
BMI, Kg/m^2^ (IQR)	26.5 (IQR 25.0 - 31.0)	26.0 (IQR 25.0 - 31.0)	39.0 (IQR 36.0 - 39.0)	<0.001
DM, n (%)	131 (54.0)	119 (54.8)	12 (92.3)	<0.001
HTN, n (%)	111 (51.6)	99 (49.0)	12 (92.3)	0.002
Smoking, n (%)	72 (33.5)	61 (30.2)	11 (84.6)	<0.001
Hyperlipidemia, n (%)	130 (60.5)	120 (57.9)	10 (76.9)	0.003
**Killip, n (%)**				**<0.001**
Killip I	125 (58.1)	125 (61.9)	0 (0.0)	
Killip II	59 (20.5)	58 (26.7)	1 (7.7)	
Killip III	32 (13.0)	23 (10.9)	9 (46.2)	
Killip IV	14 (6.5)	11 (5.4)	3 (23.1)	
**Angiographic findings (%)**				**0.48**
Non-obstructive coronary	17 (7.9)	17 (8.5)	0 (0.0)	
Single vessel disease	142(61)	136(62.7%)	6(46.2)	
Single vessel disease-ISRS	5 (2.3)	5 (2.5)	0 (0.0)	
Single vessel disease-stent thrombosis	4 (1.9)	4 (2.0)	0 (0.0)	
Two vessel disease	37 (17.2)	34 (16.8)	3 (23.1)	
Triple vessel disease	25 (11.6)	21 (10.4)	4 (30.8)	
**Culprit vessel, n (%)**				**0.18**
N/A	17 (7.9)	17 (8.4)	0 (0.0)	
LM	2 (0.9)	2 (0.9)	0 (0.0)	
LAD	112 (48.7)	101 (46.5)	11 (84.6)	
LCX	32 (13.9)	32 (14.7)	0 (0.0)	
RCA	69 (30.)	67 (30.1)	2 (15.4)	
Inotropes, n (%)	15 (7.0)	7 (3.5)	8 (61.5)	<0.001
Mechanical ventilation, n (%)	9 (4.2)	5 (2.5)	4 (30.8)	<0.001
IABP, n (%)	3 (1.4)	1 (0.5)	2 (15.4)	0.01
LOS, median (IQR)	2.0 (2.0 - 3.0)	2.0 (2.0 - 3.0)	2.0 (0.0 - 8.0)	0.74

1 : median (IQR), n (%); ^2^: Wilcoxon rank sum test, Fischer’s exact test, Pearson chi-squared test; BMI: body mass index; DM: diabetes mellitus; HTN: hypertension; LAD: left anterior descending; LCX: left circumflex; RCA: right coronary artery; IABP: intra-aortic balloon pump; LOS: length of stay; LM: left main; ISRS: In Stent ReStenosis

**Two-dimensional ECHO and laboratory findings amongst the cohort:** Table 5 below illustrates laboratory investigations for all patients and provides a comparison between survivors and non-survivors. A higher Low-Density Lipoprotein and Triglyceride (TG), with statistical significance (P-value <0.05), was noted amongst the non-survivors. The Median Left Ventricular Ejection Fraction (LVEF) was 50.23%, and non-survivors had a lower LVEF when compared to survivors. Only 12 patients (5.6%) of the 213 were noted to have LV thrombus on 2D echocardiography as seen in Table 6.

**Table 5:** laboratory investigations and comparison of survivors and non-survivors

	n	Overall, N = 230^1^	Survivors, N = 217 (94.3%)	Non- Survivors, N = 13 (5.7%)	P-value^2^
Troponin T, mean (SD)	224	1,073.6 (1,558.3)	1,045.5 (1,383.4)	1,496.4 (3,284.1)	0.46
CKMB, mean (SD)	188	76.2 (88.3)	75.7 (87.9)	86.6 (100.1)	0.58
TC, mean (SD), mmol/l	212	4.7 (1.1)	4.7 (1.1)	4.4 (1.1)	0.10
LDL, mean (SD), mmol/l	212	2.8 (0.9)	2.2 (0.9)	2.8 (1.3)	0.05
HDL, mean (SD), mmol/l	212	1.1 (0.2)	1.1 (0.2)	1.0 (0.1)	0.18
TG, mean (SD), mmol/l	212	1.8 (0.9)	1.7 (0.9)	2.5 (1.0)	0.006
HBA1C, mean (SD)%	91	6.3 (3.8)	6.3 (3.9)	6.7 (3.6)	0.79
CRP, mean (SD) mg/l	126	72.4 (111.5)	70.4 (110.0)	136.4 (158.7)	0.96
BUN, mean (SD)mmol/l	212	5.4 (2.4)	5.4 (2.4)	5.5 (2.2)	0.77
Creatinine, mean (SD) umol/l	212	79.3 (21.4)	78.9 (19.3)	84.8 (43.9)	0.41

**^1^**: mean; **^2^:** Wilcoxon rank sum tests; TC: total cholesterol; LDL: low-density lipoprotein; HDL: high-density lipoprotein; TG: triglycerides; CRP: C-reactive protein; BUN: blood urea nitrogen

**Table 6:** two-dimensional non-contrast echocardiographic findings prior to hospital discharge

Characteristic	Overall, N = 213^1^	Survivors, N = 202	Non-Survivors, N = 11	P-value^2^
LVEF, median (IQR)	50.23% (40.1 - 60.2)	50.6% (45.6 - 61.3)	30.5% (31.5 - 42.5)	<0.001
LV thrombus, n (%)	12 (5.6)	11 (7.7)	1 (16.7)	0.40

1 : median, n (%); ^2^: Wilcoxon rank sum tests. LV: left ventricle; LVEF: left ventricle ejection fraction

**Factors associated with in-hospital mortality:** the factors associated with in-hospital mortality amongst our cohort (Table 7). A BMI of above 36.2 (±5.7) (OR 1.46, 95% CI 1.17-2.10), the presence of smoking (OR 41.68, 95% CI 2.60-240.71), and the use of mechanical ventilation (OR 77.42, 95% CI 1.95-128.89) were factors associated with in-hospital mortality.

**Table 7:** factors associated with in-hospital mortality

Dependent: outcome		Survivors	Non- Survivors	OR (univariable)	p-value	OR (multivariable)	p-value
Time from chest pain to presentation (hours)		10.5 (3.0 - 24.0)	24.0 (24.0 - 36.0)	1.03 (1.00-1.06)	p=0.069	1.04 (0.97-1.14)	p=0.235
BMI		27.8 (±5.2)	36.2 (±5.7)	1.24 (1.13-1.39)	p<0.001	1.46 (1.17-2.10)	p=0.006
HTN	No	103 (99.0)	1 (1.0)		-		-
	Yes	99 (89.2)	12 (10.8)	12.48 (2.39-229.57)	p=0.016	6.49 (0.35-631.08)	p=0.301
Smoking	No	141 (98.6)	2 (1.4)		-		-
	Yes	61 (84.7)	11 (15.3)	12.71 (3.29-83.76)	p=0.001	41.68 (2.60-240.71)	p=0.025
Inotropes	No	195 (97.5)	5 (2.5)		-		-
	Yes	7 (46.7)	8 (53.3)	44.57 (12.06-185.95)	p<0.001	14.22 (0.65-650.87)	p=0.107
Mechanical ventilation	No	197 (95.6)	9 (4.4)		-		-
	Yes	5 (55.6)	4 (44.4)	17.51 (3.80-78.30)	p<0.001	77.42 (1.95-128.89)	p=0.033
IABP	No	201 (94.8)	11 (5.2)		-		-
	Yes	1 (33.3)	2 (66.7)	36.55 (3.27-823.35)	p=0.004	26.47 (0.03-130.61)	p=0.570
Triglycerides	Mean (SD)	1.7 (0.9)	2.5 (1.0)	1.94 (1.18-3.16)	p=0.007	3.33 (0.85-18.80)	p=0.097

BMI: body mass index; HTN: hypertension; IABP: intra-aortic balloon pump; OR: odds ratio

## Discussion

To our knowledge, this is the first study in the country that systematically describes clinical characteristics, interventions, and outcomes among patients presenting with STEMI for approximately a decade at a private primary PCI hospital in Tanzania.

Our results demonstrate lower in-hospital mortality for patients treated according to recommended guidelines compared to other regional studies from the Ivory Coast [17,18], Burkina Faso [19], Djibouti [20], Nigeria [21], and Mali [22]. Our morality rates are comparable to well-resourced centers in Africa [23-25], Northern America [26-30], and data from national registries of the European Society of Cardiology member countries [31,32]. The lower hospital mortality in our cohort may be attributed to our cohorts younger age, shorter time from onset to presentation, and a high rate of timely intervention and reperfusion strategies available at our institution when compared to other regions in Africa. Nonetheless, comparing outcomes across different cohorts may be confounded by many factors; including definitions of disease and outcomes, time to presentation, burden of underlying comorbidities, and availability of resources.

The mean age of our cohort is lower than that of western cohorts, but comparable to that of other African series [33]. The age range among various study populations on the African continent with STEMI has consistently been noted to be lower, with a mean age between 55 and 58 years [33]. This phenomenon may be due to various genetic factors, higher incidence of various risk factors, and socioeconomic reasons. It should also be noted that our cohort is comprised of mixed ethnicity and is not representative of the native population in Tanzania.

As noted in the INTERHEART Africa [33] multicentric study, our results continue to highlight the burden of hypertension, diabetes mellitus, obesity, and hyperlipidemia as the main cardiovascular risk factors associated with STEMI. This analysis further highlights the epidemiological transition in the country and underscores the importance of intensifying preventive medicine campaigns.

Despite the median time from onset to presentation being beyond 12 hours. The time from symptom onset to presentation was lower in our cohort compared to similar studies in the African continent [34]. This probably reflects a selection bias, as patients presenting to a private facility tend to be from a higher socioeconomic background and education level. Our facility is also located in an easily accessible area in a major urban center, and this provides an advantage. For this reason, the vast majority of the Tanzanian population is unlikely to enjoy the privilege of timely primary PCI. Delays in reperfusion therapy have been clearly linked to poor outcomes [35]. Timely intervention and Systems of care exploring thrombolysis and referral for invasive assessment have been successfully demonstrated to provide excellent outcomes in the developed world and this may be the way forward for many African healthcare systems [33-35].

The lack of PCI-capable hospitals in Africa has made the comparison of angiographic data and intervention rates challenging [34]. Our study results highlight high and timely reperfusion rates, comparable to rates documented in Northern America and Europe [36]. Large multi-center registry STEMI studies in South Africa, Ivory Coast, and Kenya have reported variable reperfusion rates ranging between 13-60% [17,34].

Numerous studies have identified low body mass index (BMI) as a predictor of poor outcomes after STEMI, with overweight and obese individuals experiencing more favorable length of stay (LOS), fewer inpatient complications, and better in-hospital, 30-day, and long-term outcomes [37-39], expanding on the concept of the “obesity paradox” which continues to remain a point of debate to date. Hypotheses that have been postulated to support the obesity paradox in STEMI patients are the higher metabolic reserve and increased development of collaterals [40]. Our results are contrary to the aforementioned reports and associate a higher BMI with an increase in in-hospital mortality and this could be related to the small size of our population. The aim of the study was beyond exploring this relationship. Nonetheless, obesity in Tanzania continues to be a growing pandemic [41,42] and a precursor of other cardiovascular risk factors such as diabetes mellitus, hypertension, and hyperlipidemia. Obesity is also a state of chronic inflammation [43] and a factor of poor functional status [41]. This collective association could account for poor hospital outcomes among patients with obesity in our cohort. It is however important to note that the obesity paradox is still a subject of ongoing research, and not all studies agree on its existence; thus, it should not overshadow the risks associated with obesity.

Our study also highlighted smoking as the main factor of in hospital mortality among patients with STEMI. Similarly, various reports have also indicated the presence of “smokers´ paradox,” suggesting favorable outcomes among smokers than non-smokers [42]. This association is intriguing, counterintuitive, and may be misleading because several epidemiological studies have clearly attributed smoking as an independent risk for atherosclerosis, heart failure, premature Atherosclerotic Cardiovascular Disease (ASCVD), and death [43]. This paradox is largely attributed to the younger age as smokers may develop an acute myocardial infarction a decade earlier than non-smokers and thus tend to have fewer cardiovascular comorbidities [42]. It has also been postulated that smoking might exert protective effects and could reduce infarct size, a strong predictor of poor outcomes among patients with STEMI [42], suggesting greater responsiveness to spontaneous or therapeutic thrombolysis. Furthermore, smoking activates the cytochrome system, an enzyme responsible for converting clopidogrel, a common antiplatelet, from its prodrug to its active form, thereby increasing its antiplatelet effect [42]. It is, however, crucial to understand that the harmful effects of smoking on the cardiovascular system outweigh any potential short-term advantages observed in a few studies. Just like the obesity paradox, the smoking paradox is still evolving and remains an area of discussion and ongoing research.

Patients with STEMI requiring mechanical ventilation during admission or hospitalization often face a more complicated clinical course. Approximately 20% of patients with STEMI typically experience respiratory impairment due to acute heart failure [44], with prior studies indicating half of this group to require invasive mechanical ventilation during their hospital stay, forming a very high-risk subgroup [44]. Our study results are parallel to published reports indicating an increase in the risk of death among those needing mechanical ventilation. Additionally, prolonged mechanical ventilation has been associated with the development of ventilator-associated pneumonia, the need for inotropes, and Intra-Aortic Balloon Pump (IABP), which collectively increase the risk of in-hospital mortality [45].

Despite key findings spanning over a decade, our study like many had several key limitations. Firstly, this was a single-center study from a private urban teaching and referral university hospital, so findings cannot be generalized nationwide. Single-center studies also risk institutional and patient selection bias. Furthermore, as expected, retrospective design is inclined to missing data. Nonetheless, we tried to extract as much as possible from medical records and databases available for consentient statistical analysis. Additionally, we were not able to accurately collect data or analyze specific timings related to outcomes among patients with STEMI, such as door-to-ECG timing and door-to-procedure timing, which have significant prognostication value.

## Conclusion

We present a decade of descriptive summary of patients who presented with STEMI at a teaching university hospital in Tanzania. Our study demonstrates a low in-hospital mortality rate of 5.7% among patients with STEMI. Cigarette smoking, obesity and the need for mechanical ventilation were predictors of poor outcomes. Large prospective, multicenter registry studies are needed to understand the magnitude of the syndrome and better highlight areas of improvement.

### What is known about this topic

- Ischemic Heart Disease (IHD) is a growing health crisis in sub-Saharan Africa (SSA) and remains the leading cause of death in adults over 60 years;
- ST-Segment Elevation Myocardial Infarction (STEMI), a severe consequence of IHD, requires rapid intervention like primary Percutaneous Coronary Intervention (PCI), which is the standard of care;
- There is limited data available on STEMI trends and in-hospital mortality in regions like Tanzania despite progress in coronary care.

### What this study adds

- This study identified cigarette smoking, obesity, and the need for mechanical ventilation as key predictors of in-hospital mortality among STEMI patients;
- The in-hospital mortality rate of the study cohort (5.7%) was lower compared to other regional studies;
- Diabetes, hypertension, and smoking were found to be more prevalent among non-survivors of STEMI in this Tanzanian cohort.
